# The YlmG protein has a conserved function related to the distribution of nucleoids in chloroplasts and cyanobacteria

**DOI:** 10.1186/1471-2229-10-57

**Published:** 2010-04-02

**Authors:** Yukihiro Kabeya, Hiromitsu Nakanishi, Kenji Suzuki, Takanari Ichikawa, Youichi Kondou, Minami Matsui, Shin-ya Miyagishima

**Affiliations:** 1 Initiative Research Program, RIKEN Advanced Science Institute, 2-1 Hirosawa, Wako, Saitama 351-0198, Japan; 2 Plant Functional Genomics Research Team, RIKEN Plant Science Center, 1-7-22 Suehiro-cho, Tsurumi-ku, Yokohama, Kanagawa 230-0045, Japan

## Abstract

**Background:**

Reminiscent of their free-living cyanobacterial ancestor, chloroplasts proliferate by division coupled with the partition of nucleoids (DNA-protein complexes). Division of the chloroplast envelope membrane is performed by constriction of the ring structures at the division site. During division, nucleoids also change their shape and are distributed essentially equally to the daughter chloroplasts. Although several components of the envelope division machinery have been identified and characterized, little is known about the molecular components/mechanisms underlying the change of the nucleoid structure.

**Results:**

In order to identify new factors that are involved in the chloroplast division, we isolated *Arabidopsis thaliana *chloroplast division mutants from a pool of random cDNA-overexpressed lines. We found that the overexpression of a previously uncharacterized gene (*AtYLMG1-1*) of cyanobacterial origin results in the formation of an irregular network of chloroplast nucleoids, along with a defect in chloroplast division. In contrast, knockdown of *AtYLMG1-1 *resulted in a concentration of the nucleoids into a few large structures, but did not affect chloroplast division. Immunofluorescence microscopy showed that AtYLMG1-1 localizes in small puncta on thylakoid membranes, to which a subset of nucleoids colocalize. In addition, in the cyanobacterium *Synechococcus elongates*, overexpression and deletion of *ylmG *also displayed defects in nucleoid structure and cell division.

**Conclusions:**

These results suggest that the proper distribution of nucleoids requires the YlmG protein, and the mechanism is conserved between cyanobacteria and chloroplasts. Given that *ylmG *exists in a cell division gene cluster downstream of *ftsZ *in gram-positive bacteria and that *ylmG *overexpression impaired the chloroplast division, the nucleoid partitioning by YlmG might be related to chloroplast and cyanobacterial division processes.

## Background

Chloroplasts arose from a bacterial endosymbiont related to extant cyanobacteria. During evolution, the majority of endosymbiont genes were lost or transferred to the nuclear genome of the eukaryotic host. Many of the nucleus-encoded proteins of cyanobacterial origin are post-translationally targeted to chloroplasts [1]. Therefore, chloroplasts retain several features similar to cyanobacteria.

Chloroplasts are never synthesized *de novo*, but proliferate by division, reminiscent of their cyanobacterial ancestor. As in bacterial division, the chloroplast division process consists of a partitioning of nucleoids (DNA-protein complexes) and fission of the two envelope membranes. The envelope membrane fission event is performed by ring structures at the division site, encompassing both the inside and the outside of the two envelopes. Consistent with the endosymbiotic theory, the division ring contains nucleus-encoded homologs of cyanobacterial division proteins, such as FtsZ [2] and ARC6 [3]. The positioning of the FtsZ ring has been shown to be regulated by cyanobacteria-derived MinD [4] and MinE [5]. Furthermore, several other fission components which were added after endosymbiosis have been identified, such as DRP5B (ARC5) [6,7], PDV1/PDV2 [8], and MCD1 [9].

On the other hand, little information is available about the molecular mechanism underlying the partitioning of chloroplast nucleoids, although earlier microscopic observations established that the localization of nucleoids changes during cell differentiation in land plants and that nucleoids are nearly equally inherited by daughter chloroplasts during chloroplast division [10,11]. In mature chloroplasts, nucleoids are observed as small particles scattered in stroma and associated with the thylakoid membrane. The condensation of nucleoids has been suggested to be maintained by the HU protein in red algae and sulfite reductase (SiR) in certain angiosperms [12-15]. In *A. thaliana*, it was reported that multiple small nucleoids form a filamentous network during chloroplast division [16]. In addition, the gene silencing of chloroplast DNA gyrase resulted in the appearance of a small number of large chloroplast nucleoids and abnormal chloroplast division in *Nicotiana benthamiana *[17]. These observations suggest a correlation between nucleoid partitioning and chloroplast division. To date, two proteins have been identified as candidates to anchor nucleoids to the thylakoid and envelope membranes. The inner envelope spanning protein PEND and the thylakoid membrane-spanning protein MFP1 were shown to bind to DNA [18-20], but both proteins are specific to angiosperms and it remains to be determined how they are involved in nucleoid partitioning.

In contrast to the chloroplast, some proteins have been shown to be involved in the partitioning of nucleoids in bacteria [21]. FtsK, a sequence-directed DNA translocase, cooperates with topoisomerase IV to decatenate the two sister chromosomes. MreB, a bacterial actin homolog, is involved in chromosome movement. SetB interacts with MreB and affects chromosome segregation [21]. However homologs of these proteins have not been found in plant genomes. At present, the molecular components/mechanisms underlying the nucleoid partitioning in the chloroplast are little understood.

In this study, we report a chloroplast division mutant that contains an aberrant network of chloroplast nucleoids from random cDNA overexpressing lines of *A. thaliana *[22]. We found that overexpression or knockdown of the previously uncharacterized gene *AtYLMG1-1 *impairs normal nucleoid partitioning, and that the overexpression also impairs chloroplast division. Similar results were obtained in the cyanobacterium *Synechococcus elongatus*. These results suggest that the partitioning of nucleoids requires YlmG. Moreover, the existence of the *ylmG *gene in the cell division gene cluster of gram-positive bacterial genomes [23], and the chloroplast division defect induced by *AtYLMG1-1 *overexpression, raises the strong possibility that nucleoid partition by YlmG might be related to chloroplast division.

## Results

### Isolation of *A. thaliana *chloroplast division mutants from FOX lines

Several proteins required for chloroplast division have been identified and characterized by both forward and reverse genetics. These studies yielded the identification of FtsZ [2], MinD [4], MinE [5], DRP5B/ARC5 [6,7], ARC3 [24], ARC6 [3], PDV1/PDV2 [8], and MCD1 [9].

Although analyses by using conventional loss-of-function mutants have contributed to the identification of these chloroplast division proteins, certain key chloroplast division genes may as yet still be uncovered, e.g. because of the lethality of a given mutation or the functional redundancy provided by duplicate genes. In order to identify any such unidentified chloroplast division genes by an alternative approach, we searched for genes that affect on chloroplast division when the genes are overexpressed.

**Figure 1:**
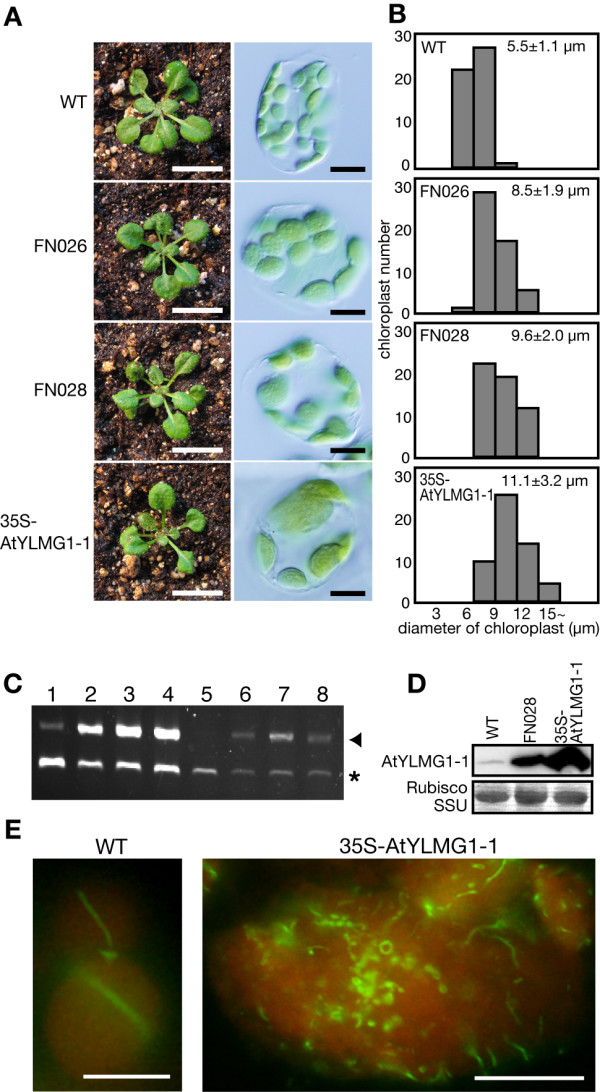
**Phenotypes of the *AtYLMG1-1 *overexpressers**. (A) Three-week-old seedlings of the FOX line (FN026 and FN028), and plants with a *35S *promoter-At3g07430 transgene (35S-AtYLMG1-1). Chloroplasts in single leaf mesophyll cells of FN026, FN028, and the *35S*-*AtYLMG1-1 *transgenic plant. Bars = 10 mm (left) and 10 μm (right). (B) The average of the chloroplast diameter is shown in each graph along with the standard deviation. n = 50. (C) Levels of the *AtYLMG1-1 *transcript in the wild type, FOX lines and the *35S*-*AtYLMG1-1 *transgenic plants. Transcript levels were analyzed by RT-PCR in the wild type (lane 1 and 5), FN026 (lane 2 and 6), FN028 (lane 3 and 7), and the *35S*-*AtYLMG1-1 *transgenic plants (lane 4 and 8). A micro litter (lane 1-4) or 0.1 μl (lane 5-8) of reverse-transcription product was used as the PCR template. *GAPDH *was used as the quantitative control. Triangle indicates the RT-PCR products of *AtYLMG1-1*, and asterisk indicates that of *GAPDH*. (D) Levels of the AtYLMG1-1 protein in the wild type, FOX line, and the *35S*-*AtYLMG1-1 *transgenic plants. Total proteins extracted from 3-week-old seedling of the wild type (WT), FOX line (FN028), and the *35S*-*AtYLMG1-1 *transgenic plants (35S-AtYLMG1-1) were analyzed with the anti-AtYLMG1-1 antibodies raised against a peptide fragment of AtYLMG1-1. Fifty micrograms of proteins were loaded in each lane. The Rubisco small subunit (Rubisco SSU) was detected by Coomassie brilliant blue (CBB) staining as the quantitative control. (E) Localization of FtsZ in the wild type and the *AtYLMG1-1 *overexpresser. Localization of FtsZ2-1 in mesophyll cells was examined under immunofluorescence microscopy. The green fluorescence shows the localization of FtsZ2-1 and the autofluorescence of chlorophyll is depicted in red. Bars = 5 μm.

To this end, we screened ~15,000 T2 plants of the full-length cDNA overexpresser (FOX) gene-hunting lines of *A. thaliana *[22] by microscopic observation of mesophyll cell chloroplasts. As a result, we isolated 18 mutant lines that contained a smaller number and larger size of chloroplasts than the wild type. Of these mutants, two lines (FN026 and FN028) contained cDNA of the same gene (At3g07430) downstream of the cauliflower mosaic virus (CaMV) 35S promoter (Figure 1A). All the F1 progeny, after crossing FN026 and FN028 with the wild type, displayed the mutant phenotype, indicating that the phenotype occurs in a dominant manner. When At3g07430 was overexpressed in the wild-type plants by a newly constructed 35S-At3g07430 transgene, the resulting plant contained chloroplasts significantly larger than the wild type (two times on average, Figure 1B). Reverse transcriptase-polymerase chain reaction (RT-PCR) analyses confirmed that the level of At3g07430 transcript was increased in the FN026, FN028, and 35S-At3g07430 lines (Figure 1C). In order to examine the protein level of At3g07430, we prepared antibodies against At3g07430. On immunoblots, the antibodies detected a band of ~20 kDa, which is consistent with the predicted size (23 kDa) of the At3g07430 protein [the transit peptide was predicted by the TargetP http://www.cbs.dtu.dk/services/TargetP/ and the predicted transit peptide was omitted for calculation of the molecular mass](Figure 1D). Immunoblot analyses confirmed that At3g07430 protein was over-produced in the FN028 and 35S-At3g07430 lines and showed that the At3g07430 protein level of the 35S-At3g07430 line was higher than that of the FN028 line (Figure 1D). Therefore there is a correlation between the level of At3g07430 protein (Figure 1D) and the chloroplast size (Figure 1A and 1B). In contrast to the protein level, the At3g07430 transcript level of the FN028 line was higher than that of the 35S-At3g07430 line. This is likely due to the difference between inserted transgenes. The transgene in the FN028 line contains full-length At3g07430 cDNA whereas the 35S-At3g07430 line contains no 5'-UTR. The presence or absence of the 5'-UTR probably affects on the efficiency of the translation of At3g07430 protein.

Although the function of the At3g07430 protein has not been determined, the database [The Arabidopsis Information Resource (TAIR); http://www.arabidopsis.org/] indicates that a homozygous T-DNA insertion mutant of this gene (CS24080) resulted in an embryonically defective phenotype. Because a BLAST search showed that At3g07430 is homologous to the bacterial YlmG protein which is of unknown function (for details, see below), we named the gene *AtYLMG1-1*.

In order to explore how chloroplast division is impaired in the *AtYLMG1-1 *overexpresser, we compared the localization of FtsZ in the overexpresser and the wild type. Immunofluorescence microscopy using an anti-FtsZ2-1 antibody [9] showed FtsZ localization at the chloroplast division site in the wild type (Figure 1E). In contrast, the localization of FtsZ was perturbed in the overexpresser, with fragmented filaments, small rings, and dots observed in almost all of the chloroplasts (Figure 1E). Therefore overexpression of AtYLMG1-1 perturbs the FtsZ ring formation and consequently impairs chloroplast division.

### Phylogenetic relationships in the YlmG family

**Figure 2:**
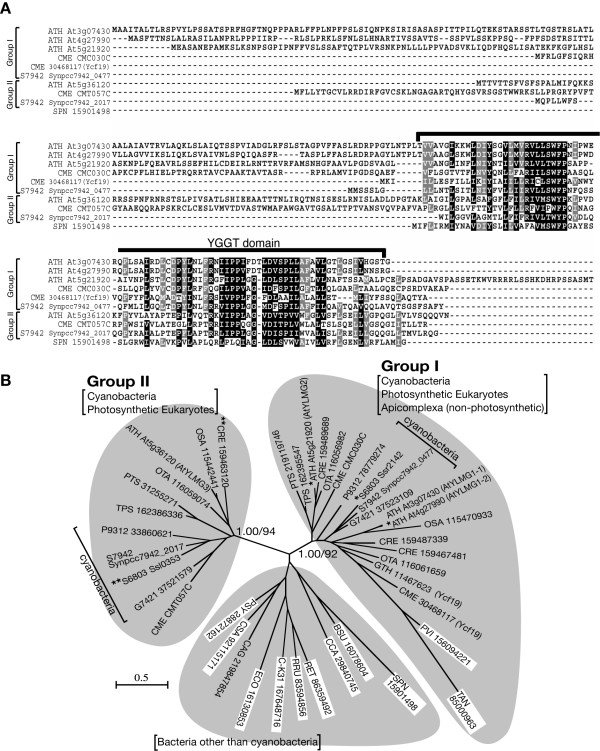
**Phylogenetic relationships in the YlmG family of proteins**. (A) Amino acid sequence alignment of the YlmG family. The amino acid sequences were collected from the National Center for Biotechnology Information database. The alignment includes the YlmG family of proteins of *A. thaliana *(ATH), the red alga *Cyanidioschyzon merolae *(CME), the cyanobacteria *S. elongatus *PCC7942 (S7942), and *S. pneumoniae *(SPN). The locus IDs or GI numbers of the sequences are indicated with the name of the species. (B) Phylogenetic tree of the YLMG family. The tree shown is the maximum-likelihood tree constructed by the PHYML program [48]. The numbers at the selected nodes are posterior probabilities by the Bayesian inference (left) and local bootstrap values provided by the maximum-likelihood analysis (right). The tree includes proteins of photosynthetic eukaryotes; *A. thaliana *(ATH), *Oryza sativa *(OSA), *Chlamydomonas reinharditii *(CRE), *Ostreococcus tauri *(OTA), *C. merolae *(CME), *Thalassiosira pseudonana *(TPS), and *Phaeodactylum tricornutum *(PTR), apiconplexa; *Plasmodium vivax *(PVI) and *Theileria annulata *(TAN), cyanobacteria; *Synechocystis *sp. PCC 6803 (S6803), *S. elongatus *PCC7942 (S7942), *Gloeobacter violaceus *PCC 7421 (G7421), and *Prochlorococcus marinus *str. MIT 9312 (P9312), other bacteria; *Escherichia coli *(ECO), *Bacillus subtilis *(BSU), *Streptococcus pneumoniae *(SPN), *Chlamydophila caviae *(CCA), *Rhizobium etli *(RET), *Rhodospirillum rubrum *(RRU), *Caulobacter *sp. K31 (C-K31), *Chloroflexus aggregans *(CAG), *Chromohalobacter salexigens *(CSA), and *Pseudomonas syringae *(PSY). The locus IDs or GI numbers of the sequences are shown with the name of the species. White boxes indicate non-photosynthetic organisms. * indicates proteins whose gene disruptants showed no effects on the activity of the photosystems, while ** indicates proteins whose gene disruptants reduced the photosystem activity [29,30,32]. Posterior probabilities and bootstrap values for all branches are shown in Additional file 1.

BLAST and PSI BLAST searches along with sequence alignment indicated that AtYLMG1-1 is homologous to the bacterial YlmG proteins and the chloroplast-encoded Ycf19 of unknown function (Figure 2A; see also Additional file 1). The YlmG protein contains a putative membrane spanning YGGT domain (according to the name of *E. coli *gene *yggT *of unknown function). In addition, the searches showed that YlmG-related sequences are conserved in and specific to bacteria and plastid-carrying eukaryotes. In the genome of *A. thaliana*, four homologs of YlmG were identified (At3g07430, At4g27990, At5g21920, and At5g36120).

In the genomes of gram-positive bacteria, the *ylmG *gene locates downstream of the cell division gene *ftsZ *in the *dcw *(division and cell wall) cluster in the order *ylmD, ylmE, ylmF, ylmG, ylmH*, and *divIVA *[23]. Of these genes, *ylmF *(*sepF*) [25-27] and *divIVA *[28] have been shown to be involved in cell division in several bacterial lineages. These results raise the possibility that the YlmG protein is also involved in cell division, although inactivation of *ylmG *in *Streptococcus pneumoniae *did not in fact result in any apparent effect on cell division [23]. On the other hand, the *Chlamydomonas reinhardtii *YlmG homolog, CCB3, has been implicated in cytochrome *b*_6 _maturation, based on the result that CCB3 complemented the defect in cytochrome *b*_6 _maturation in *ccb *mutants [29]. In addition, disruption of the ortholog in *Synechocystis *sp. PCC6803 impaired photosynthetic activity [30].

In order to clarify the relationship of YlmG homologs in bacteria and eukaryotes, we performed phylogenetic analyses (Figure 2B). The analyses indicate that oxygenic-photosynthetic organisms (i.e. cyanobacteria and chloroplast-carrying eukaryotes) have two distinct families (Group I and II) with high support values (local bootstrap value by the maximum likelihood method/posterior probability by Bayesian inference, 92/1.00 for group I, 94/1.00 for Group II). Group II contains only proteins of oxygenic photosynthetic organisms, consistent with the reports suggesting a relationship between CCB3 and photosynthesis. In contrast, group I, to which AtYLMG1-1 belongs, contains proteins of apicomplexan parasites, which have non-photosynthetic plastids (apicoplasts) acquired by a red algal secondary endosymbiosis [31]. This pattern of gene distribution suggest that both group I and II of cyanobacterial *ylmG *were transferred to the nuclear genomes of plants by a primary endosymbiosis of the chloroplast, and that group II, but not group I, was lost in parallel with the loss of photosynthetic activity in the ancestor of apicomplexans. Given this scenario and the existence of *ylmG *in non-photosynthetic bacteria, it is suggested that group I (including AtYLMG1-1) and bacterial YlmG (other than cyanobacterial group II) are not related to photosynthesis. Supporting this suggestion, recent studies have shown that the inactivation of the members of group I (*A. thaliana*, At4g27990 and At5g21920; *Synechocystis *sp. PCC 6803, *ssr2142*) had no effect on the photosynthesis [30,32].

The phylogenetic analyses categorized four *Arabidopsis *YLMGs into the Group I and II and showed a very close relationship between AtYLMG1-1 and At4g27990. We therefore named At4g27990 AtYLMG1-2, At5g21920 AtYLMG2, and At5g36120 AtYLMG3, respectively.

### The relationship between AtYLMG1-1 and nucleoid structure

**Figure 3:**
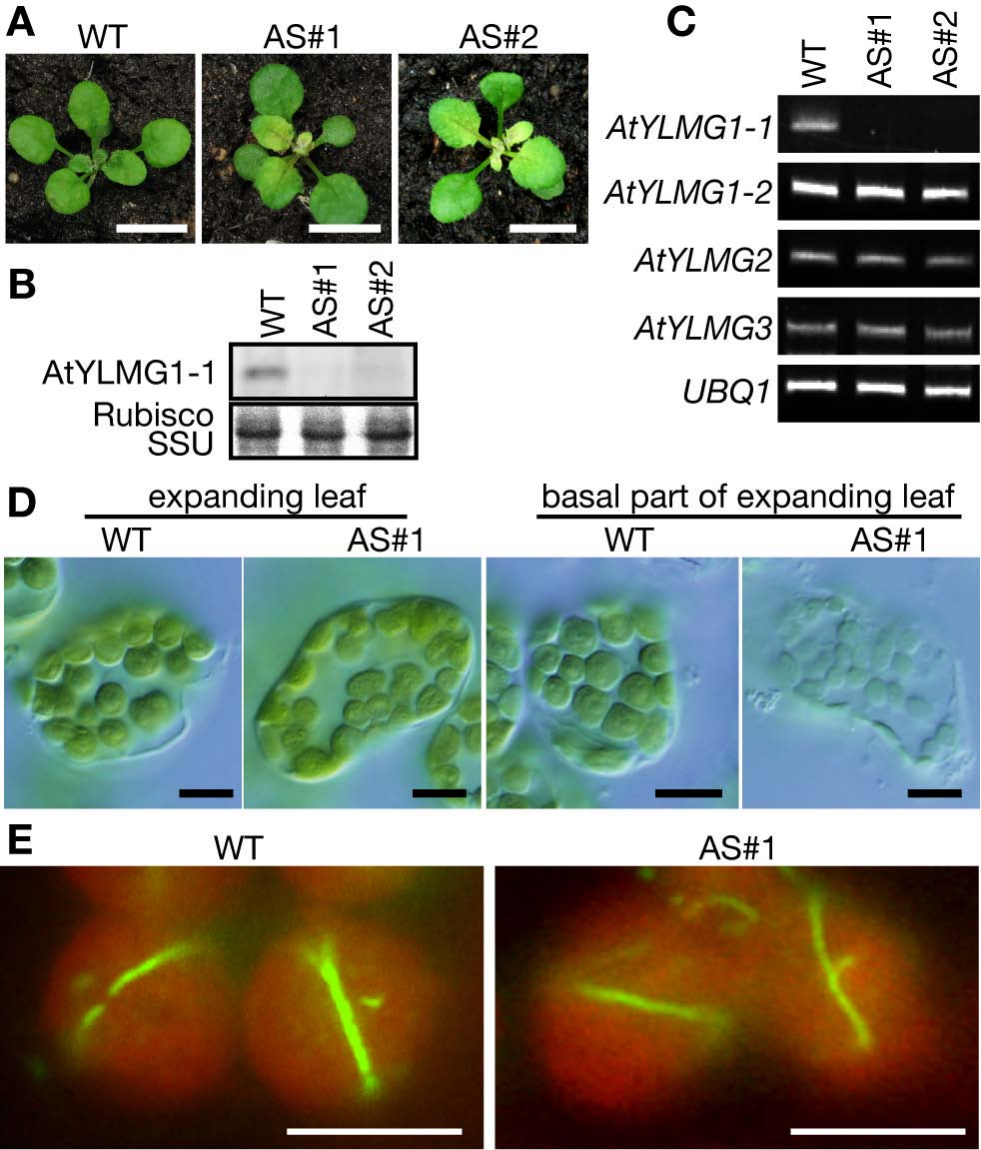
**Phenotype of the *AtYLMG1-1 *knockdown plant**. (A) Three-week-old seedlings of the wild type (WT) and the *AtYLMG1-1 *knockdown line (AS#1 and AS#2). Bars = 10 mm. (B) Levels of AtYLMG1-1 protein in the wild type and the two *AtYLMG1-1 *knockdown lines. Total proteins extracted from the wild type (WT) and the two *AtYLMG1-1 *knockdown lines (AS#1 and AS#2) were analyzed with the anti-AtYLMG1-1 antibodies. Fifty micrograms of proteins were loaded in each lane. The Rubisco small subunit (Rubisco SSU) was detected by CBB staining as the quantitative control. (C) Levels of *AtYLMG1-1 *and other *YLMG *gene transcripts in the wild type and two independent *AtYLMG1-1 *knockdown lines (AS#1 and AS#2). The levels of *AtYLMG1-1*, *AtYLMG1-2*, *AtYLMG2*, and *AtYLMG3 *transcripts were analyzed by RT-PCR in the wild type, AS#1, and AS#2. *UBQ1 *was used as the quantitative control. (D) Chloroplasts in leaf mesophyll cells of the wild type and the *AtYLMG1-1 *knockdown line. Chloroplasts in expanding leaf cells and the basal part of expanding leaf cells of the wild type (WT) and the *AtYLMG1-1 *knockdown line (AS#1) are shown. Bars = 10 μm. (E) Localization of FtsZ in the wild type and the *AtYLMG1-1 *knockdown line (AS#1). Localization of FtsZ2-1 in mesophyll cells was examined by immunofluorescence microscopy. The green fluorescence shows the localization of FtsZ2-1 and the autofluorescence of chlorophyll is depicted in red. Bars = 5 μm.

The chloroplast division defect observed above was caused by AtYLMG1-1 overexpression, and we were not able to obtain a homozygote of the *AtYLMG1-1 *T-DNA insertion mutant (CS24080) as reported in the TAIR database. To further examine the function of AtYLMG1-1, we expressed the antisense RNA in the wild-type plant to knockdown *AtYLMG1-1 *(Figure 3A). Immunoblot analysis confirmed that the AtYLMG1-1 protein was hardly detectable in two antisense lines (Figure 3B). The result further confirmed that the band detected by the antibodies is AtYLMG1-1 protein and showed that the antibodies do not cross-react with AtYLMG1-2 protein. RT-PCR analyses confirmed that there was a decrease of the *AtYLMG1-1 *transcript level in the antisense line and no effect on the accumulation of other *YLMG *transcripts (Figure 3C). In the antisense line, young emerging leaves and the basal part of expanding leaves exhibited a pale-green phenotype. As leaves matured, the leaf color shifted to green, with no obvious difference compared to the wild-type leaves (Figure 3A). These phenotypes were not observed in the *AtYLMG1-1 *overexpresser (Figure 1A).

Then the morphology of chloroplasts in the AtYLMG1-1 antisense line was observed under microscopy (Figure 3D). In contrast to the overexpresser, the shape and size of chloroplasts in expanding leaves were similar to those in the wild type. In the basal part of expanding leaf of the antisense line, chloroplasts were pale and smaller than those of the wild type (Figure 3D). We compared the localization of FtsZ in the antisense line and the wild type (Figure 3E). In the antisense line, FtsZ localization was observed at the chloroplast division site, as in the wild type. These results suggest that the knockdown of AtYLMG1-1 had no effect on chloroplast division.

**Figure 4:**
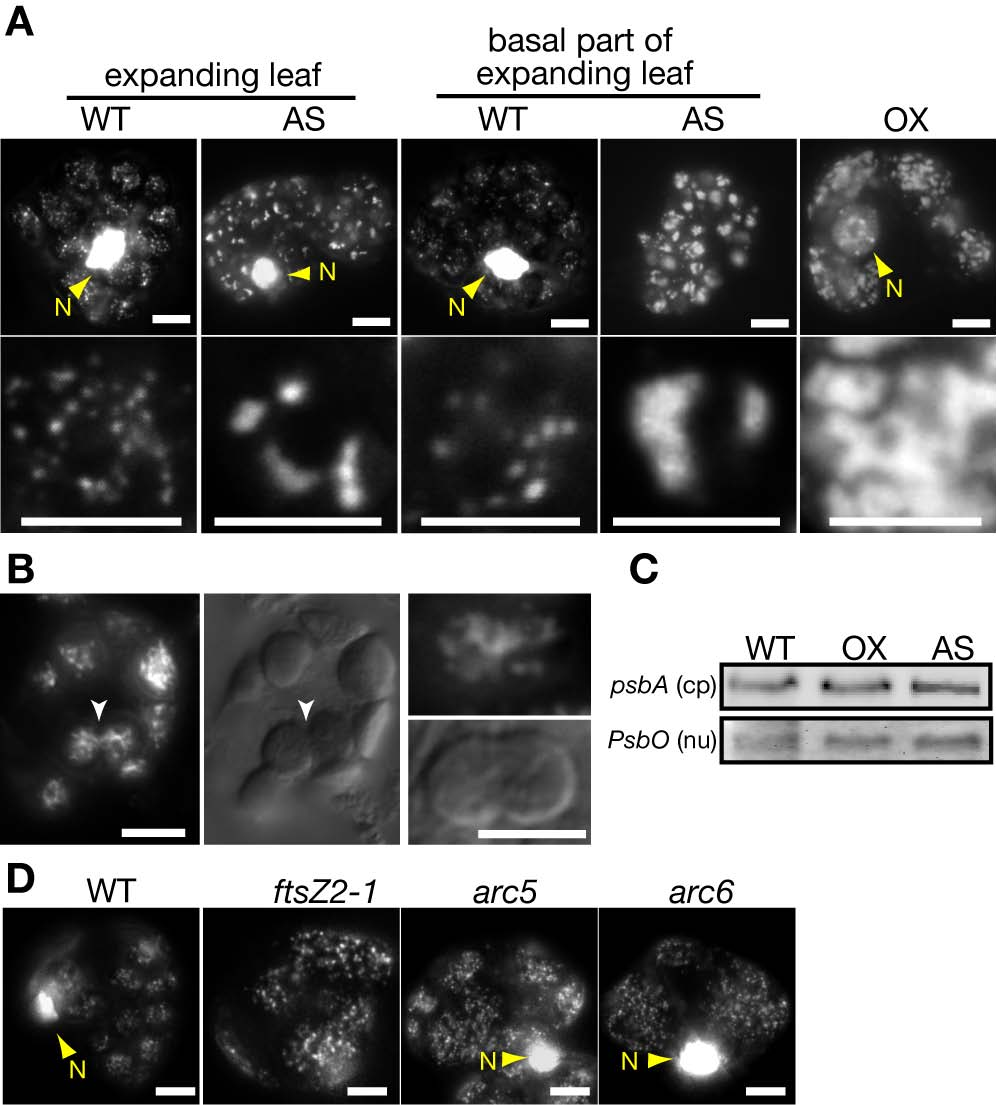
**Effects of the overexpression and knockdown of *AtYLMG1-1 *on the morphology of the chloroplast nucleoids**. (A) Morphology of the chloroplast nucleoids in the overexpresser and the knockdown lines. Expanding leaf or the basal part of expanding leaf cells of the wild type (WT), the *AtYLMG1-1 *knockdown line (AS), and the *AtYLMG1-1 *overexpresser (OX) were stained with DAPI. The white portion indicates DAPI fluorescence showing the localization of DNA. Nuclei (N) are also observed in some panels. Magnified images are also shown in the lower panels. Bars = 5 μm. All images were obtained with the same exposure time. (B) Morphology of the nucleoids in dividing chloroplasts. Young emerging leaves of the wild type were stained with SYBR GREEN I. The white portion indicates the SYBR GREEN I fluorescence showing the localization of DNA. Arrowheads indicate dividing chloroplasts. Other dividing chloroplasts are also shown in the right panels. Bars = 10 μm. (C) Comparison of the quantity of chloroplast DNA by DNA-blot analysis. Total genome DNA of the wild type (WT), the *AtYLMG1-1 *overexpresser (OX), and the *AtYLMG1-1 *knockdown line (AS) was extracted and then was digested with *Hin*dIII. Three micrograms of digested DNA were loaded in each lane. Chloroplast DNA (cp) was detected with a *psbA *probe and nuclear DNA (nu) was detected with a *PsbO *probe. Nuclear DNA was detected as the quantitative control. (D) Morphology of the chloroplast nucleoids in *ftsZ2-1*, *arc5*, and *arc6 *mutants. Mature leaves of the *ftsZ2-1*, *arc5*, and *arc6 *mutants were stained with DAPI. The white portion indicates DAPI fluorescence showing the localization of DNA. Bars = 5 μm. All images were obtained with the same exposure time.

Although the knockdown did not impair envelope division, the existence of *ylmG *in the *dcw *cluster of gram-positive bacteria suggests the gene product may be involved in bacterial division. To examine whether AtYLMG1-1 is required for a process other than envelope fission that is related to chloroplast division, we observed chloroplast nucleoids in the antisense line and the wild type. By 4', 6-diamidino-2-phenylindole (DAPI) staining, nucleoids were observed as small particles dispersed in mature chloroplasts of the wild type (Figure 4A). In contrast, nucleoids were concentrated in a few large structures in both the tip and basal part of the expanding leaves of the antisense line. When the AtYLMG1-1 overexpresser was examined, the nucleoids were observed as irregular networks. These networks of nucleoids are similar to those in dividing chloroplasts, although the fluorescent intensity by DAPI-staining was higher in the overexpresser than in the wild type (Figure 4B, [16]). In both the antisense line and the overexpresser, DNA gel blot analyses showed that the amount of chloroplast DNA compared to the nuclear DNA is similar to that of the wild type (Figure 4C). These results suggest that knockdown or overexpression of AtYLMG1-1 does not affect the replication of chloroplast DNA, but does affect the morphology of nucleoids.

To examine whether abnormal structure of nucleoids causes the chloroplast division defect in the AtYLMG1-1 overexpresser or chloroplast division defects result in the abnormal nucleoids, we observed nucleoids in *bona fide *chloroplast division (envelope division) mutants. In contrast to the AtYLMG1-1 overexpresser, the morphology of chloroplast nucleoids in *ftsZ2-1, arc5*, and *arc6 *mutants was similar to the wild type (Figure 4D). Taken together, the above results suggest that AtYLMG1-1 is required for the proper distribution of nucleoids in chloroplasts. It is also suggested that abnormality of the nucleoid structure is not due to a chloroplast division defect, but rather, the abnormal nucleoids induced by AtYLMG1-1 overexpression might be a cause of the chloroplast division defect.

### Localization of AtYLMG1-1

**Figure 5:**
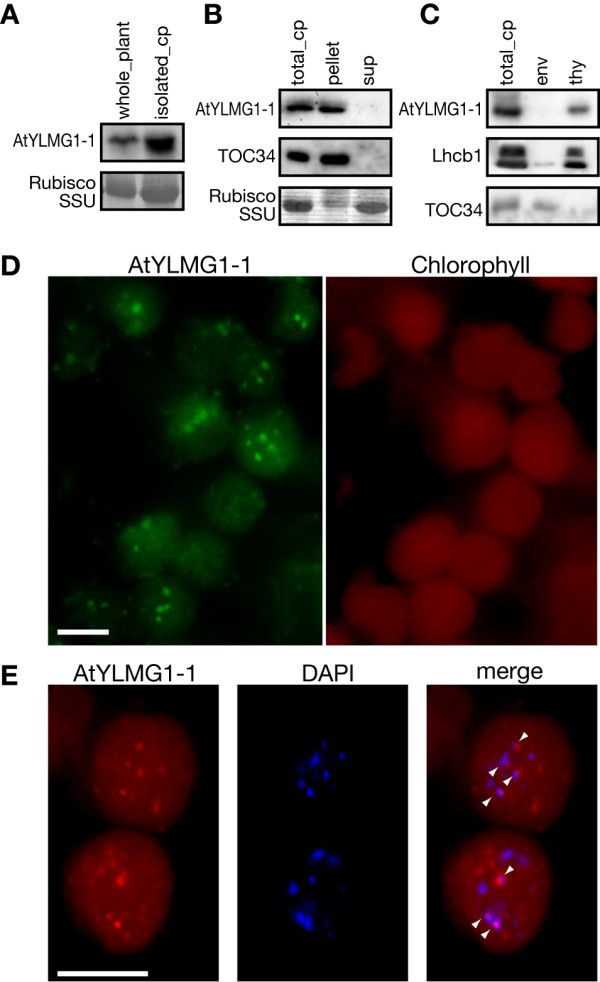
**Localization of the AtYLMG1-1 protein**. (A) Immunoblot analysis showing the chloroplast localization of AtYLMG1-1. Total proteins extracted from whole plants and isolated chloroplasts (cp) from the wild type were analyzed with the anti-AtYLMG1-1 antibodies. Fifty micrograms of protein were loaded in each lane. The Rubisco small subunit (Rubisco SSU) was detected by CBB staining as the quantitative control. (B) Localization of AtYLMG1-1 in the chloroplast. Chloroplasts were lysed in hypotonic solution and separated into pellet and supernatant fractions by centrifugation. The total chloroplast protein (total cp), pellet (pellet), and supernatant (sup) fractions were analyzed. TOC34 was detected as a marker of the membrane protein and the Rubisco small subunit was detected as a marker of the stromal protein. (C) Localization of AtYLMG1-1 in the chloroplast membranes. Isolated chloroplasts from the wild type were lysed and separated into thylakoid and envelope membranes. Proteins of the total chloroplast (total cp), the envelope fraction (env), and the thylakoid fraction (thy) were examined with the anti-AtYLMG1-1 antibodies. Lhcb1 was detected as a marker of the thylakoid protein and TOC34 was detected as a marker of the envelope protein. (D) Localization of AtYLMG1-1 examined by immunofluorescence microscopy. Isolated chloroplasts from the wild type were immunostained with the anti-AtYLMG1-1 antibodies. The green fluorescence indicates the localization of AtYLMG1-1 and the red shows the chlorophyll fluorescence. Bar = 5 μm. (E) Relationship between AtYLMG1-1 puncta and chloroplast nucleoids. Isolated chloroplasts were immunostained with the anti-AtYLMG1-1 antibodies and counterstained with DAPI. The red indicates the localization of AtYLMG1-1 and the blue is DAPI fluorescence showing the localization of DNA. A merged image is also shown. Arrowheads indicate the overlap between the AtYLMG1-1 puncta and nucleoids. Bar = 5 μm.

In order to obtain insight into whether AtYLMG1-1 directly affects the distribution of nucleoids, we examined the localization of AtYLMG1-1. Immunoblot analyses showed that AtYLMG1-1 was enriched in the isolated chloroplasts as compared with the whole plant protein (Figure 5A). When the chloroplasts were lysed in hypotonic solution, AtYLMG1-1 was detected in the membrane fraction (pellet), as was the membrane protein TOC34, suggesting that AtYLMG1-1 is a chloroplast membrane protein (Figure 5B), as predicted in the database ARAMEMNON http://aramemnon.uni-koeln.de/. Further fractionation showed that AtYLMG1-1 is exclusively associated with the thylakoid membranes, as is Lhcb1 (Figure 5C).

We further examined the intrachloroplast localization of AtYLMG1-1 by immunofluorescence microscopy using AtYLMG1-1 antibodies. The fluorescent signals were detected on the punctate structures dispersed in chloroplasts of the wild-type leaves (Figure 5D). These results, together with the results of the immunoblotting, indicate that AtYLMG1-1 localizes in the puncta on thylakoid membranes. Comparison of the immunofluorescence and the DAPI fluorescence showed that some of the AtYLMG1-1 puncta co-localize with a subset of nucleoids (Figure 5E).

### Effect of overexpression and gene disruption of *ylmG *in the cyanobacterium *S. elongatus*

**Figure 6:**
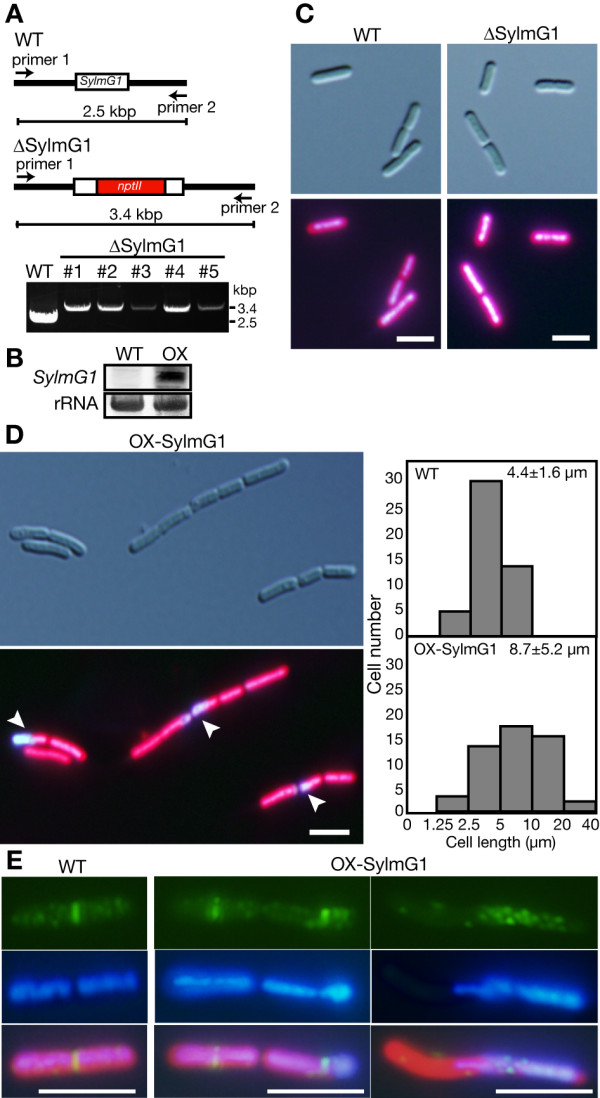
**Effects of disruption or overexpression of *SylmG1 *in *Synechococcus elongatus***. (A) Gene disruption of *SylmG1*. Genotypic character of the wild type (WT) and kanamycin-resistant mutants (lines #1-5) which were subjected to PCR analysis using primer 1 (5'-TGACGGACTTCTTCGACCAGATG-3') and primer 2 (5'-ATTGAACCGCGTTGGGACAAGG-3'). A 0.9-kb *nptII *gene was inserted into *SylmG1 *locus by homologous recombination. The insertion of the *nptII *gene was confirmed by PCR using the primer set indicated in the diagram. (B) Overexpression of *SylmG1*. Total RNA (3 μg) from exponential cells (OD_730 _= 0.4) of the wild type or spectinomycin-resistant mutants (OX) was subjected to RNA-blot analysis with the *SylmG1 *specific probe. (C) Phenotype of the *SylmG1 *disruptant. Nucleoids of the wild type (WT) and the *SylmG1 *disruptant (Δ*SylmG1*) were stained with DAPI. Cells in the exponential phase were stained with DAPI and the images were obtained with the same exposure time. The blue is DAPI fluorescence showing the localization of DNA, and the autofluorescence of chlorophyll is red. Bars = 5 μm. (D) Nucleoids of the *SylmG1 *overexpresser (OX-SylmG1). The image was obtained by the same procedure as (c). The distribution patterns of the cell length of the wild type (WT) and the *SylmG1 *overexpresser, measured in the exponential phase, are shown in the histograms. The average of the cell length is shown in each graph along with the standard deviation. n = 50. Bar = 5 μm. (E) Relationship between the distribution of nucleoids and the localization of FtsZ in the wild type and the *SylmG1 *overexpresser. Localization of FtsZ was examined by immunofluorescence microscopy. The green fluorescence shows the localization of FtsZ. The blue is DAPI fluorescence which shows the localization of DNA, and the autofluorescence of chlorophyll is red. Merged images are also shown at the bottom. Bars = 5 μm.

Knockdown of *AtYLMG1-1 *had no effect on chloroplast division, but the lack of an evident chloroplast division phenotype might be due to the existence of two other genes related to *AtYLMG1-1 *(AtYLMG1-2 and AtYLMG2, shown in Figure 2B). Our phylogenetic analyses indicated that cyanobacterial species have only single genes encoding group I and group II YlmG proteins, respectively. Therefore, in order to examine whether the group I *ylmG *is involved in bacterial cell division, and whether the function of *ylmG *is conserved between chloroplasts and cyanobacteria, we examined the effects of group I *ylmG *(ORF ID; Synpcc7942_0477, *SylmG1*) disruption and overexpression in *Synechococcus elongatus*. We disrupted the *SylmG1 *gene by homologous recombination and insertion of a kanamycin-resistant gene into the *SylmG1 *locus (Figure 6A). Because cyanobacteria have multiple genomes [33,34], PCR was used to determine whether the mutations were completely or incompletely segregated. In the wild type, a 2.5 kbp DNA fragment that contains *SylmG1 *gene was amplified. In contrast, the 2.5 kbp DNA fragment was not detected and a 3.4 kbp DNA fragment was detected in the five independent kanamycin-resistant transformants (Figure 6A). These results indicate that the 0.9 kbp *nptII *gene cassette was integrated into the *SylmG1 *genomic locus and that the mutation was completely segregated. To overexpress *SylmG1*, the bacterial consensus II promoter and *SylmG1 orf *fusion was integrated into a neutral site of *the S. elongatus *genome [35]. RNA gel blotting indicated that *SylmG1 *was overexpressed in the transformants (Figure 6B).

To examine the effect of disruption and overexpression of the *SylmG1 *gene on cell division as well as nucleoid structure, cells in the exponential phase were stained with DAPI and observed under microscopy. Although the shape and length of the Δ*SylmG1 *cells were similar to the wild type, the intensity of DAPI fluorescence was higher in Δ*SylmG1 *(Figure 6C). However, the amount of total DNA extracted from the same number of cells did not differ between the wild type and Δ*SylmG1 *(Δ*SylmG1 */wild type, 1.03 ± 0.01). These results suggest that nucleoid compaction occurred in Δ*SylmG1*. On the other hand, *SylmG1 *overexpressers frequently contained cells significantly longer than the wild-type cells (two times longer on average, Figure 6D), suggesting that cell division is partially impaired in the overexpresser. In addition, abnormal distribution of nucleoids was observed in the overexpresser (Figure 6D and 6E middle panel) and ~2% cells exhibited extremely biased segregation of nucleoids during cell division (Figure 6E right panel). These results suggest that the overexpression of *SylmG1 *impairs nucleoid segregation during cell division. To further examine how cell division is impaired in the overexpresser, we examined FtsZ localization by immunofluorescence microscopy using anti-FtsZ antibodies. The antibodies detected the FtsZ ring at the mid-cell position in the wild type (Figure 6E). In the *SylmG1 *overexpressers, the FtsZ rings had a tendency to be biased towards the side of the cell to which nucleoid density was biased (Figure 6E middle panel). In addition, a diffuse but higher concentration of FtsZ localization was observed around the region where nucleoid density was biased (Figure 6E right panel). These results suggest that SYlmG1 is required to maintain normal nucleoid structure, and that the FtsZ localization might be related to the nucleoid partitioning by YlmG.

## Discussion

In this study, we screened chloroplast division defective mutants in the *A. thaliana *FOX-hunting system. The purpose of using the FOX line was to identify genes in which the disruption is lethal or which does not exhibit an obvious phenotype due to existence of redundant genes. As a result, we found that AtYLMG1-1 overexpression impairs the normal partitioning of chloroplast nucleoids and chloroplast division. On the other hand, we could not obtain a homozygote of the *AtYLMG1-1 *T-DNA insertion mutant (CS24080), and the heterozygote did not display any difference from the wild type. Therefore, this study is a good example of an effective use of the FOX line.

The YlmG family of proteins is widely distributed in bacteria and plastid-carrying eukaryotes. Thus far, there are two different candidate functions put forward. The presence of *ylmG *in the bacterial *dcw *cluster implies that the gene product might be related to cell division [23]. On the other hand, analyses of mutant phenotypes in plants and cyanobacteria suggest that YlmG is required for normal activity of the photosystems [29,30,32]. However, in *Synechocystis *sp. PCC6803, which has two YlmG-related genes, disruption of *ssl0353 *reduced the activity of the photosystems, while disruption of *ssr2142 *had no effect [30]. In *A. thaliana*, which has four homologs ofthe YLMG, mutation in At4g27990 and At5g21920 did not affect the activity of the photosystems [30,32]. Our phylogenetic analysis indicates that oxygenic-photosynthetic organisms have two different groups of YlmG. Group II contains proteins the mutations of which impair the photosystems, while group I contains proteins the mutations of which do not affect photosystems. This distribution is consistent with the fact that species of apicomplexa, which have non-photosynthetic plastids, possess group I but not group II. Utilizing genetic approaches, here we have shown that the group I proteins, AtYLMG1-1 and SylmG1, are required for the normal partitioning of nucleoids.

Immunofluorescence microscopy revealed that AtYLMG1-1 localizes in punctate structures on the thylakoid membranes which are adjacent to a subset of nucleoids (Figure 5E). Therefore we further investigated whether AtYLMG1-1 has a DNA-binding ability, but we could not get recombinant YlmG proteins by expression in *E. coli *due to the lethality of the YlmG overexpresser. Although the deduced AtYLMG1-1 amino acid sequence contains no predicted DNA/RNA-binding motif, the isoelectric point of putative AtYLMG1-1 (the deduced transit peptide was removed) is 10.9. The high isoelectric point is characteristic of a large number of DNA-binding proteins, such as eukaryotic histones [36], bacterial HU [37], ribonucleases [38], and bZIP transcription factors [39], and is known to be required for electrostatic interaction with DNA. Therefore, an AtYLMG1-1 punctate structure in close proximity to a nucleoid may interact electrostatically with the nucleoid, thus an anchoring the nucleoid to the thylakoid membrane.

In *A. thaliana*, chloroplasts in the *AtYLMG1-1 *knockdown line contained a small number of enlarged nucleoids, while in the *AtYLMG1-1 *overexpresser, nucleoids were observed as filamentous networks (Figure 4A). These opposite effects suggest that YlmG is required for the filamentation of nucleoids, and probably also for partitioning. Given that the AtYLMG1-1 punctate structures exist adjacent to a subset of nucleoids (Figure 5E), it is possible that the partitioning of nucleoids is gradually executed from nucleoids connected to the AtYLMG1-1. Further time-lapse observation will clarify this hypothesis. In this regard, however, stable expression of AtYLMG1-1-GFP by the AtYLMG1-1 promoter did not successfully complement the lethal phenotype of the T-DNA insertional mutant. Even when the AtYLMG1-1-GFP was expressed by *AtYLMG1-1 *promoter in the wild type, a fluorescent signal was not detected, suggesting that the expression level of AtYLMG1-1 is relatively low. Therefore, other approaches will be required for further analyses.

As shown in FtsZ, MinD, MinE, ARC6, GC1, and ARC3 [3,24,40-43], alteration in the stoichiometry among these proteins impairs normal chloroplast division. Because overexpression of AtYLMG1-1 impairs FtsZ localization and the chloroplast division (Figure 1E), the alternation of the AtYLMG1-1 level might disturb the stoichiometric relationship among the chloroplast division machinery. However, the AtYLMG1-1 knockdown did not impair chloroplast division unlike *bona fide *chloroplast division proteins. In addition, AtYLMG1-1 localizes in puncta on thylakoid membranes, it therefore is unlikely that the AtYLMG1-1 level directly affects on the stoichiometry among the *bona fide *chloroplast division proteins.

Previous study [16] and our own observation (Figure 4B) showed that nucleoids exhibit a filamentous network during chloroplast division. The chloroplast nucleoids in the AtYLMG1-1 overexpresser were similar to the nucleoid structure during chloroplast division, although the fluorescent intensity of DAPI staining was higher in the overexpresser than the wild type. Furthermore, FtsZ localization and chloroplast/cell division were impaired in both *A. thaliana *and *S. elongatus *by overexpression of *ylmG*. In the *ylmG *overexpresser of *S. elongatus*, FtsZ localized predominantly to the area where nucleoids were biased (Figure 6E). These results imply that nucleoid partitioning by YlmG might be related to the formation of the FtsZ ring. In several lineages of bacteria, the FtsZ ring assembly is blocked in the vicinity of nucleoids in order to partition the genome properly into daughter cells by a nucleoid occlusion mechanism [44]. However, previous observations showed that the typical nucleoid occlusion mechanism does not apparently function in cyanobacteria [25]. Unlike other bacteria containing a single copy of the genome, cyanobacteria and chloroplasts contain multicopies of the genome. At present, little information is available about the relationship between nucleoids and the division of chloroplasts and cyanobacteria. Further study of the function of YlmG should provide significant insights into this relationship.

## Conclusions

Our results show that overexpression of AtYLMG1-1 protein causes formation of filamentous structure of chloroplast nucleoids, and that knockdown of AtYLMG1-1 causes the aggregation of nucleoids. In addition, the overexpression impairs FtsZ ring formation and chloroplast division. Overexpression and deletion of the *ylmG *gene in the cyanobacterium *Synechococcus elongatus *displayed defects similar to that in *A. thaliana*, suggesting that the function of the YlmG protein, which is engaged in the proper distribution of nucleoids, is conserved between cyanobacteria and chloroplasts. AtYLMG1-1 localizes in small puncta on thylakoid membranes, which are structures connected with a subset of nucleoids. The YlmG-containing punctate structures on the thylakoid membrane required for the proper distribution of nucleoids and the proper distribution of nucleoids is likely required for both normal FtsZ ring formation and chloroplast division.

## Methods

### Growth of organisms

*A. thaliana *(Col-0) was used as the wild type. *A. thaliana *seeds were surface-sterilized, sown on Murashige and Skoog (MS) plates, and stratified at 4°C for 2 days in the dark before germination. Plants were grown in controlled-environment chambers with 16 h of light (100 μmol/m^2^s) and 8 h of dark at 20°C. Seedlings were transferred onto soil and were grown in the controlled-environment chambers.

*Synechococcus elongatus *was grown in BG-11 medium [45] in 50 ml flasks on a rotary shaker or 1.2% agar plates at 30°C in continuous light (100 μmol/m^2^s). Growth of cells in the liquid cultures was measured by determining OD_730_.

### Isolation of chloroplast division mutants in the FOX library

T2 seeds of the *A. thaliana *FOX lines were germinated and grown on MS plates for 3 weeks. Tips from expanding leaves were put on a glass slide without fixation, covered with a cover slip, and smashed gently. Samples were observed with Nomarski differential interference contrast optics.

To identify the inserted *35S*-cDNA in the FOX lines, the insertion was amplified by PCR using primers 5'-GTACGTATTTTTACAACAATTACCAACAAC-3' and 5'-GGATTCAATCTTAAGAAACTTTATTGCCAA-3', and then sequenced by a primer 5'-CCCCCCCCCCCCD (A or G or T)-3'.

### Construction and generating transgenic *A. thaliana*

For overexpression of *AtYLMG1-1*, a genomic region containing *AtYLMG1-1 orf *was amplified by primers 5'-ATGTCTAGAATGGCCGCCATTACAGCTCTC-3' (the *Xba*I site is underlined) and 5'-ATGGAGCTCCGTTTCAACAAAACCATTAGC-3' (the *Sac*I site is underlined). For expression of antisense *AtYLMG1-1 *gene, a genomic fragment was amplified by primers 5'-ATGTCTAGATCACAGAGATCTCTAATGGCA-3' (the *Xba*I site is underlined) and 5'-AGTGAGCTCTCTTCAACAGGCGGAATAAC-3' (the *Sac*I site is underlined). These amplified products were digested with *Xba*I and *Sac*I and were inserted between *Xba*I and *Sac*I sites of pBI121 vector. Above constructs were transferred to *Agrobacterium tumefaciens *GV3101 and introduced into *A. thaliana *plants as described [46]. Transformants were selected on the MS medium containing 30 mg/L kanamycin and T2 plants were used for further analyses.

### Construction and generating transgenic *S. elongatus*

For targeted disruption of *SylmG1 *gene (ORF ID; Synpcc7942_0477), a unique restriction site (*Xba*I site) was added to the genomic fragment containing *SylmG1 *by overlap-extension PCR. We amplified a 100 bp of *SylmG1 orf *franked by an 850 bp upstream sequence by primers 5'-CCGCGATCGGCTCTCGCGTGATTGCCAGCG-3' and 5'-CATTCTAGAGGAACCAGCTCAGTAAGACGC-3' (the *Xba*I site is underlined). A 200 bp of *SylmG1 orf *franked by an 850 bp downstream sequence was amplified by primers 5'-ACTGAGCTGGTTCCTCTAGAGAGCAGTCAGTTCATGCTGAT-3' and 5'-ACGGTGGCGATGAGCACGGCTACACCGACT-3' (the *Xba*I site is underlined). These two amplified fragments were mixed and fused by PCR using primers 5'-CCGCGATCGGCTCTCGCGTGATTGCCAGCG-3' and 5'-ACGGTGGCGATGAGCACGGCTACACCGACT-3'. The fused fragment was cloned into pGEM-T easy (Promega). An *orf *of *nptII*, which confers resistance to kanamycin, was amplified by primers 5'-ATGTCTAGAAGCTATGACCATGATTACGAA-3' and 5'-ATGTCTAGAAAGTCAGCGTAATGCTCTGCC-3' (the *Xba*I site is underlined), digested with *Xba*I, and inserted into *SylmG1 orf *in which an *Xba*I site was introduced as above. A construct in which the *nptII *cassette was inserted in the same orientation was used for gene disruption. Genotypic character of the wild type and kanamycin-resistant mutants (lines #1-5) which were subjected to PCR analysis with primers 5'-TGACGGACTTCTTCGACCAGATG-3' and 5'-ATTGAACCGCGTTGGGACAAGG-3'.

For overexpression of *SylmG1*, a DNA fragment containing *SylmG1 orf *was amplified by primers 5'-ATGCCCGGGGACAGATTTATTGGACGGTGA-3' and 5'-ATGCCCGGGCAAGCGGAGCTCTATCACGAA-3' (the *Sma*I site is underlined). The amplified product was digested by *Sma*I and inserted into *Sma*I site of the pTY1002 vector, which contains a bacterial consensus II promoter, *aadA *gene which confers resistence to spectinomycin, and *S. elongatus *neutral site (position is 2577767-2578661 and 2578658-2580657) [35].

Above constructs were transformed into the wild type cells. Transformants were selected on BG-11 plates containing 15 mg/L kanamycin or 10 mg/L spectinomycin. For the *SylmG1 *disruption, homologous recombination and segregation were confirmed by PCR using primers 5'-TGACGGACTTCTTCGACCAGATG-3' and 5'-ATTGAACCGCGTTGGGACAAGG-3'. Overexpression of *SylmG1 *transcript was confirmed by a RNA gel blot analysis using digoxigenin-labeled *SylmG1 *specific probe.

### Semi-quantitative RT-PCR

DNA-free total RNA (1 μg) was reverse-transcribed using Primescript (Takara) with oligo(dT)_15 _primer. PCR was performed by using primers 5'-CACCGAGAAGTCAACAGCTCGGTCATCGAC-3' and 5'-TCAAGTCTTCCAATTTCTACCCAGTGCTGC-3' for *AtYLMG1-1*, 5'-CCTCAACATATATAACACCATC-3' and 5'-GACAGGTTCAGGTCATAGAAG-3' for At5g21920, 5'-TATCTGAACACTCCGTTGACGGTA-3' and 5'-CAAAGATAAACGGAATACGATC-3' for At4g27990, 5'-GCAATGGGAAGCAGTGGTGG-3' and 5'-GGGAGAAGAGACGGGTTTCG-3' for *GAPDH*, and 5'-GGCCAAGATCCAAGACAAAG-3' and 5'-GTTGACAGCTCTTGGGTGAA-3' for *UBQ1*.

### Phylogenetic analyses

Deduced amino acid sequences of YlmG homologs encoded by the 41 genes were collected by BLAST searches against public databases. The sequences were aligned by Clustal X 2.0 [47], manually refined and 82 amino acid residues corresponding to the YGGT domain were used for the phylogenetic analyses. Maximum likelihood trees were constructed PHYML [48] based on the WAG model with the discrete gamma model for site-heterogeneity (8 categories with 100 replications). Bayesian inference was performed with the program MrBayes version 3.1.2 [49] using the WAG matrix assuming a proportion of invariant positions and four gamma-distributed rates. For the MrBayes consensus trees, 1,000,000 generations were completed with trees collected every 100 generations.

### Microscopy

For observation of chloroplast size and number, tips from expanding leaves were cut and fixed by 3.5% glutaraldehyde for 1 h at room temperature and then incubated in 0.1 M Na_2_-EDTA pH 9.0 for 15 min at 50°C. Samples were analyzed with Nomarski differential interference contrast optics.

For observation of chloroplast nucleoids, leaves were cut into small fragments and then digested in 2% cellulase RS (Yakult), 1% macerozyme (Yakult), 550 mM sorbitol, and 5 mM MES-KOH pH5.8 at 25°C for 30 min. Resulting protoplasts were stained by 1 μg/ml DAPI in TAN buffer (0.5 M sucrose, 0.5 mM EDTA, 1.2 mM spermidine, 7 mM 2-mercaptoethanol, 0.4 mM phenylmethyl-sulfonyl fluoride, 20 mM Tris-HCl, pH 7.5) or SYBR Green I (Invitrogen) after fixation with 1% glutaraldehyde.

Localization of FtsZ in *A. thaliana *expanding leaves was examined by immunofluorescence microscopy using an anti-AtFtsZ2-1 antibodies as described [9]. Localization of FtsZ in *S. elongatus *was examined by immunofluorescence microscopy using an anti-Anabaena FtsZ antibodies (AS07217, Agrisera) as described [25] except for Can Get Signal (Toyobo) was used for the antibody reactions.

Localization of AtYLMG1-1 in chloroplasts of expanding leaves was examined using rabbit anti-AtYLMG1-1 antibodies which were raised against two synthetic peptides (FASLRDRPPGYLNT and TEKSTARSSTLTGS). Isolated chloroplasts were fixed by 2% paraformaldehyde dissolved in TAN buffer. For antigen retrieval, fixed chloroplasts were treated by 20 μg/ml proteinaseK in TE buffer containing 0.5% Triton X-100 at 37°C for 10 min. Chloroplasts were washed twice with PBS. After blocking with 3% skim milk in PBS for 30 min, the samples were incubated for 16 h at 4°C with the anti-AtYLMG1-1 antibodies diluted 1:200 in Can Get Signal. Chloroplasts were then washed twice with 3% skim milk in PBS and then incubated with anti-rabbit IgG conjugated with AlexaFluor 488 (Invitrogen) for 2 h at room temperature. To observe chloroplast DNA, samples were stained with 1 μg/ml DAPI.

### DNA-blot analysis

Total DNA were extracted from ~3 weeks plants grown on the MS medium. Three-micrograms DNA were digested with *Hin*dIII and the DNA fragments were separated on 0.8% agarose gels. The restriction fragments were transferred to Hybond-N+ membranes. Hybridization and detection were carried out as described using digoxigenin-labeled DNA probes [50]. A psbA probe was amplified with the primers 5'-TGCATAAGAATGTTGTGCTCAGCC-3' and 5'-CTACTTCTGCAGCTATTGGATTGC-3', and a PsbO probe was amplified with the primers 5'-CAATCGTGCGATTTCACAGCCACTC-3' and 5'-TTCTCTTCCAAGTTGTGTCGTCTCC-3'.

### Immunoblot analyses

Intact chloroplasts were isolated from ~3 weeks plants grown on the MS medium as described [20]. To obtain insoluble (pellet) and soluble (sup) fraction of chloroplasts, intact chloroplasts were resuspended in hypotonic buffer (10 mM Tris-HCl, pH 8.0 and 1 mM EDTA), incubated for 15 min on ice, and then centrifuged at 15,000 *g *for 30 min at 4°C. To separate the thylakoid and the envelope membranes, intact chloroplasts were resuspended in Tricin buffer (50 mM Tricin pH 7.6 and 5 mM MgCl_2_), frozen at -80°C and thawed. The chloroplast lysate was separated in a sucrose density gradient (lysate/0.6 M/1.0 M/1.2 M/1.5 M) by a centrifugation at 113,000 *g *for 1 h at 4°C. The envelope membrane (at the 0.6 M/1.0 M interface) and the thylakoid membrane (at the 1.2 M/1.5 M interface) were collected.

Immunoblot analyses were performed as described [50] except that 17.5% polyacrylamide gel was used and 10 mM CAPS buffer containing 10% methanol were used for the transfer onto PVDF membrane. AtYLMG1-1 was detected with the anti-AtYLMG1-1 antibodies (1: 1,000). Anti-TOC34 (AS07 238, Agrisera) and Anti-Lhcb1 (AS01 004, Agrisera) were used at dilutions of 1: 5,000 and 1: 2,000, respectively. After detection of these proteins, the Rubisco small subunit was detected by staining the same membranes with CBB.

## Abbreviations

CaMV: cauliflower mosaic virus; CBB: Coomassie brilliant blue; DAPI: 4': 6-diamidino-2-phenylindole; dcw: division and cell wall; FOX: full-length cDNA overexpresser; RT-PCR: reverse transcriptase-polymerase chain reaction.

## Authors' contributions

HN, KS, and SM designed the screening of *A. thaliana *FOX lines and HN and KS screened the mutants and identified inserted cDNA by supervision of YKo, TI, and MM. YKa designed this study, produced the AtYLMG1-1 overexpressing and knockdown lines, produced the SYlmG1 overexpressing and gene disrupted lines, characterized the phenotypes of mutant lines, and drafted manuscript. All authors read and approved the final manuscript.

## Supplementary Material

Additional file 1 **Phylogenetic relationships in the YlmG family of proteins**. Posterior probabilities (left) and bootstrap values (right) for all branches (Figure 2) are shown here. - indicates the bootstrap values and posterior probabilities less than 50 and 0.9, respectively. Click here for file
