# Supplementary material for: The YlmG protein has a conserved function related to the distribution of nucleoids in chloroplasts and cyanobacteria
Source: BMC Plant Biol. 2010 Apr 2;10:57. doi: 10.1186/1471-2229-10-57 (PMC2923531; doi:10.1186/1471-2229-10-57)
Supplement: Additional file 1 — Phylogenetic relationships in the YlmG family of proteins. Posterior probabilities (left) and bootstrap values (right) for all branches (Figure 2) are shown here. - indicates the bootstrap values and posterior probabilities less than 50 and 0.9, respectively. [file 1471-2229-10-57-S1.PDF]

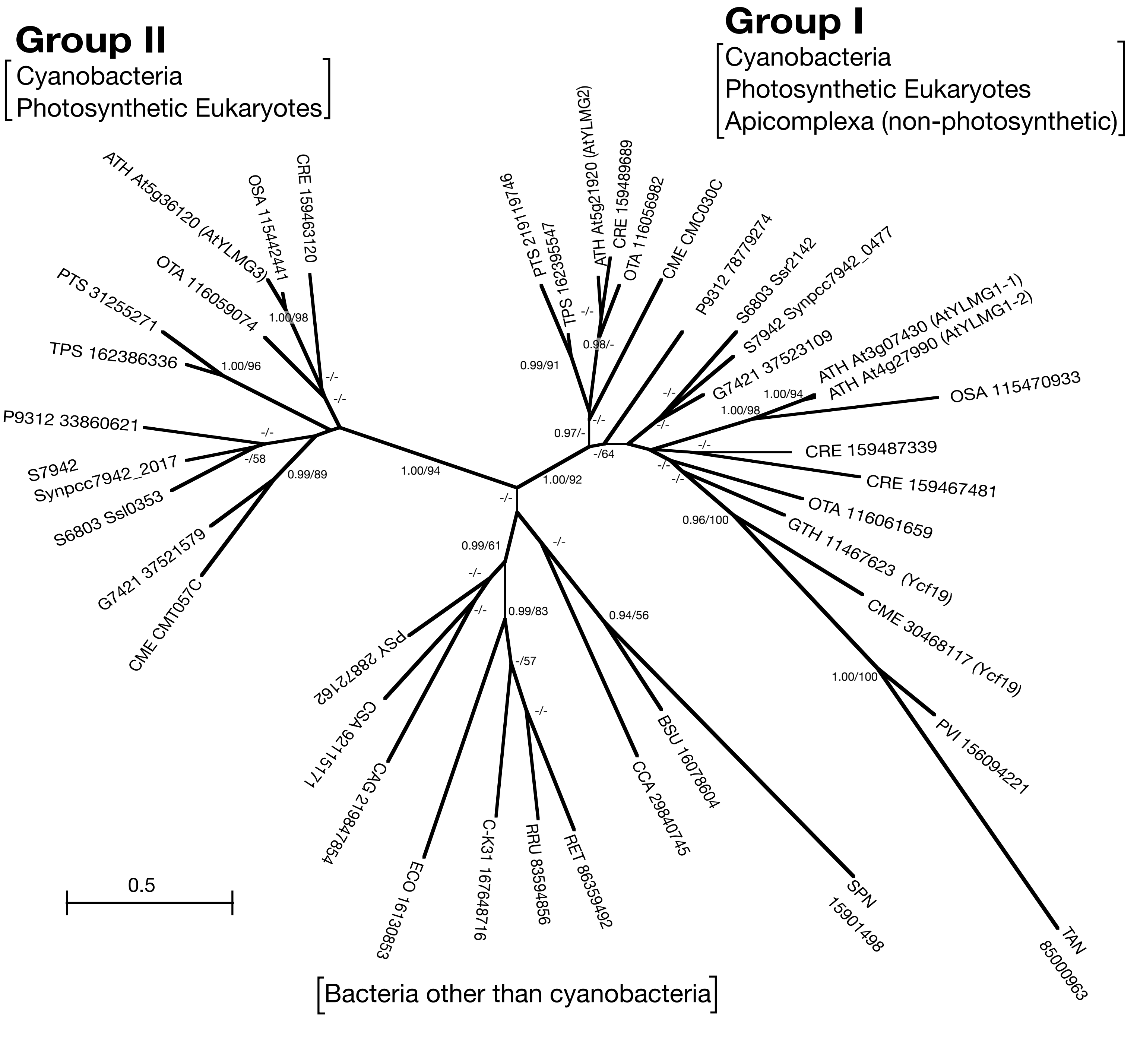
Group II
Cyanobacteria
Photosynthetic Eukaryotes
Group I
Cyanobacteria
Photosynthetic Eukaryotes
Apicomplexa (non-photosynthetic)
ATH At5g36120 (AtYLMG3)
OSA 115442441
CRE 159463120
OTA 116059074
PTS 31255271
TPS 162386336
P9312 33860621
S7942
Synpcc7942_2017
S6803 Ssl0353
G7421 37521579
CME CMT057C
PTS 219119746
TPS 162395547
ATH At5g21920 (AtYLMG2)
CRE 159489689
OTA 116056982
CME CMC030C
P9312 78779274
S6803 Ssr2142
S7942 Synpcc7942_0477
G7421 37523109
ATH At3g07430 (AtYLMG1-1)
ATH At4g27990 (AtYLMG1-2)
OSA 115470933
CRE 159487339
CRE 159467481
OTA 116061659
GTH 11467623 (Ycf19)
CME 30468117 (Ycf19)
PVI 156094221
TAN 85000963
SPN 15901498
BSU 16078604
CCA 29840745
RET 86359492
RRU 83594856
C-K31 167648716
ECO 16130853
CAG 21984785 4
CSA 92115171
PSY 28872162
1.00/98
1.00/96
0.99/89
-/58
1.00/94
1.00/92
0.99/91
0.98/-
0.97/-
-/64
1.00/94
1.00/98
0.96/100
1.00/100
0.94/56
0.99/61
0.99/83
-/57
0.5
Bacteria other than cyanobacteria
